# Fraxin in Combination with Dexamethasone Attenuates LPS-Induced Liver and Heart Injury and Their Anticytokine Activity in Mice

**DOI:** 10.1155/2023/5536933

**Published:** 2023-09-11

**Authors:** Nada Sahib Shaker, Hayder Bahaa Sahib

**Affiliations:** ^1^Al-Nahrain University, College of Medicine, Pharmacology Department, Baghdad, Iraq; ^2^Al-Nahrain University, College of Pharmacy, Baghdad, Iraq

## Abstract

**Background:**

Cytokine storm syndrome (CSS) is a major cause of morbidity and mortality in people suffering from hyperinflammatory status, which diverse etiological factors, including pathogens, therapeutic interventions, malignancies, and autoimmune disorders, can instigate. Since there is limited research on the antioxidant properties of fraxin and no studies have investigated its potential as an anticytokine storm agent, it is important to note that most studies have primarily focused on proinflammatory cytokines such as IL-1*β*, IL-6, and TNF*α* during cytokine storm. However, little research discusses the role of chemokines, particularly IL-8, during cytokine storms. Therefore, further investigation is warranted into the role of fraxin as an anticytokine storm agent and the involvement of IL-8 in cytokine storms. The present study examines the preventive efficacy of fraxin and the combination of fraxin and dexamethasone (FD) in mitigating lipopolysaccharide-induced systemic inflammation in mice caused by *Escherichia coli*, 055: B5.

**Methods:**

Five groups of ten mice were randomly assigned: LPS only group (5 mg/kg, intraperitoneally i.p.), control (normal saline N.S. 1 ml/kg, i.p.), concentrations were selected based on previous literature, fraxin (120 mg/kg, i.p.), dexamethasone (5 mg/kg, i.p.), fraxin + dexamethasone (FD) (60 mg/kg + 2.5 mg/kg, i.p.), administered one hour before LPS injection (5 mg/kg,i.p.), animals were euthanized next day, and interleukin-8 (IL-8) was quantified in serum using an enzyme-linked immunosorbent assay. The liver and heart tissues underwent histopathological analysis to assess morphological changes. For data analysis using ANOVA and Tukey post hoc tests, the Kruskal–Wallis and Mann–Whitney *U* tests were employed to analyze the histological results.

**Results:**

A significant decline in IL-8 levels was recorded in the treatment groups almost to the same degree (*p* < 0.001), and the percentage of inhibition of IL-8 for fraxin, dexamethasone, and FD was 93%.92.4%, and 93%, respectively, compared to the LPS-only group. Histopathological scores were significantly reduced in liver and heart tissue (*P* < 0.05).

**Conclusions:**

All interventions used in this study significantly reduced interleukin-8 (IL-8) levels and reduced LPS-induced liver and cardiac damage. The combination (FD) did not result in an evident superiority of either agent. More research is required to identify the possible usefulness of these agents in treating hyperinflammatory diseases, such as cytokine storms, in future clinical practice.

## 1. Introduction

Cytokine storm syndrome (CSS) is a state of excessive release of proinflammatory cytokines in which there is excessive inflammation, requiring the administration of anticytokine and antiinflammatory drugs. The immunopathology of COVID-19 is necessary to be understood, and in most critical cases, a huge increment of proinflammatory cytokines has been recorded, causing CSS and activating acute respiratory distress (ARD) toward multiorgan destruction [1].

Inflammation is tissues' natural biological response to chemical and mechanical stimuli or infectious agents, most often instigated by a diverse range of bacterial species. Pathogenic conditions such as chronic infections and the inflammatory response can cause notable detriments to the host, namely, lipopolysaccharide (LPS), a fundamental constituent of the external membrane of Gram-negative bacteria that has been widely used to develop an inflammatory model [2].

Following the introduction of LPS in experimental animals, several endotoxic effects, such as fever, leukopenia, leukocytosis, thrombocytopenia, disseminated intravascular coagulation, and hemodynamic alterations, may terminate in fatal shock [3].

The initial step in the classical interactions between lipopolysaccharide (LPS) and host cells involves binding LPS to the lipopolysaccharide binding protein (LBP). Subsequently, this complex forms a bond with a receptor complex comprising CD14, MD2, and toll-like receptor 4 (TLR4), thereby initiating a signaling cascade that elicits the secretion of proinflammatory cytokines [4].

However, previous studies have confirmed that macrophages and endothelial cells can also internalize lipopolysaccharide (LPS) via routes not dependent on toll-like receptor 4 (TLR4). After being internalized, lipopolysaccharide (LPS) can form a bond with the cytosolic receptor caspase-4/5 in humans and the corresponding caspase-11 in mice. Caspases-4/5 form oligomers and initiate the assembly of the inflammasome-3, which consists of the nucleotide-binding domain, leucine-rich family, and pyrin domain-containing protein [5].

This assembly then leads to the activation of inflammatory caspase-1, leading to the subsequent release of interleukin-1*β*. Caspases-4/5 are known to initiate the activation of perforin gasdermin D and purinergic receptor P2X7, hence leading to cell lysis and pyroptosis induction. Pyroptosis is a prominent contributor to the inflammatory response and impairment of the pulmonary endothelial barrier in cases of sepsis [6]. Neutrophil granulocytes represent the predominant cellular constituents of the innate immune system. The adequate activation of these cellular entities is fundamental to effectively eliminating pathogens. Upon exposure to a potent chemoattractant, neutrophils undergo activation and subsequently migrate to the site of inflammation, thus constituting the primary cellular defense mechanism against such insults [7]. Interleukins, such as IL-8, are frequently used as a diagnostic and prognostic indicator for conditions related to infection (specifically septic conditions) and other types of inflammation (such as traumatic conditions). Given the potential importance of IL-8-mediated neutrophil activation and its associated mechanisms in systemic inflammation, there is significant scientific and clinical interest in understanding these processes. Specifically, such insights may provide valuable information on possible pharmacological targets to regulate an excessive inflammatory response resulting from IL-8 actions [8]. In recent times, there have been numerous revelations about natural products found within plants that exhibit unique structural attributes and demonstrate diverse biological activities. The use of these therapeutic agents has furnished numerous valuable primary compounds for the development of new drugs. Polyphenols, particularly flavonoids, have been found to manifest physiological and pharmacological effects comprising antioxidant, antiinflammatory, antidiabetic, antibacterial, antitumor, and neuroprotective properties [9]. The field of drug discovery is based on phytochemicals as the primary focus for developing new small-molecule chemical entities. Plant-derived natural products are recognized as a significant and crucial class of compounds that contribute to developing established and emerging pharmacological treatments. Contemporary reports indicate that many pharmaceutical companies have reduced their emphasis on exploring potential therapeutic agents derived from natural sources [10]. Fraxin (isolated from Cortex Fraxini) is a 7, 8-dihydroxy-6-methoxy coumarin, and 8-D glucopyranoside is characterized by a wide range of activities, including antioxidant, analgesic, antimicrobial, antiviral, and immunomodulatory [11]. Fraxin has been found to exhibit significant pharmacological versatility, showing dual protection against oxidative stress and inflammation in various variables [12]. As such, it can be hypothesized that fraxin may facilitate a fundamental role in attenuating interleukin-8 release and conferring protection against LPS-induced harm in the murine population [13]. The current research aims to evaluate Fraxin's hepatoprotective and cardioprotective properties and combine it with dexamethasone in mice exposed to lipopolysaccharide (LPS). The study investigated the influence of the said substances on interleukin-8 levels.

## 2. Materials and Methods

### 2.1. Chemicals and Reagents

The chemicals and reagents utilized in this study are listed in Table 1.

### 2.2. Animals

*Swiss Albino BALB/c* mice (fifty males, 20–25 g, 7–8 weeks) were used (animals purchased from the Drug Quality Control and Research National Center, Animal House Facility, Iraq), randomly housed five in each cage with a 12-hour light/dark cycle, and mice were acclimated for a week and kept in a pathogen-free place with a temperature of 23°C ± 2°C and a humid atmosphere. Enough food and water were available all the time for all the rodents. The Animal Ethics Committee of Al-Nahrain University, College of Pharmacy, accepted the experimental protocol under issue number Nah. Co. Pha.12.

### 2.3. Study Design

Following a period of adaptation lasting one week, a total of 50 male mice were divided into five groups at random allocation, each consisting of 10 mice, as shown in Table 2; all interventions were administered one hour before induction intraperitoneally (ip) with LPS (5 mg/kg). [14].

### 2.4. Serum Collection and Quantification of Cytokine Levels

After 24 hours, after lipopolysaccharide administration (LPS), mice were subjected to anesthesia using a high dose of inhalational diethyl ether. Blood samples were then collected from the jugular veins and centrifuged at a speed of 2857 × *g* for 10 minutes for serum separation. The serum was carefully transferred to Eppendorf tubes and stored at −20°C for cytokine measurement. To ensure consistency and accuracy, it is recommended to do measurements in triplicate. Quantification of the proinflammatory cytokine, interleukin-8, in mouse serum, was performed using an enzyme-linked immunosorbent assay (ELISA) kit provided by Shanghai YL Biont, China, according to the instructions provided by the supplier. The IL-8 measurement was conducted utilizing the biotin double antibody sandwich technique. The optical density (OD) was measured at a wavelength of 450 nm. The standard curve is subjected to linear regression analysis using standardized concentrations and optical density (OD) values. Cytokine concentrations were quantified and expressed in units of nanograms per liter (ng/L).

### 2.5. Histopathological Analysis

Animals were sacrificed using a high dose of inhalational diethyl ether and cervical dislocation for 24 hours. After LPS injection, organs were dissected and fixed with 10% formalin and then embedded in paraffin for slide preparation. The tissues were dehydrated in 50%, 70%, 80%, and 95% alcohol for 10 min each and 100% ethanol for 30 min, then soaked in low-melting paraffin wax, cut into 4 mm sections, deparaffinized in xylene, and rehydrated with alcohol. H&E staining is used to visualize 4 *μ*m histological sections. A blinded professional pathologist with specific organ codes analyzed samples.The parameters for scoring histological liver damage were identified as follows: the extent of degradation of the liver lobules, the infiltration of inflammatory cells, the appearance of hemorrhage, and the necrosis of hepatocytes. The degree of hepatocellular injury was evaluated by microscopic examination at 400x magnification using the following categorizations: (1) minimal damage (0–25%), (2) mild-to-moderate damage (25–50%), (3) significant damage (50–75%), and (4) severe damage (75–100%). [15]. The degree of heart injury was evaluated based on the following: score (0) indicates no damage, score (1) indicates damage <25% of the total area, score 2 indicates damage (25–75%) of the total area, and score (3) indicates severe damage more than 75% of the total area [16]. The Zeiss Imager M2 microscope (Carl Zeiss Microimaging) equipped with an Axio Cam MRc CCD camera (Carl Zeiss) was used to photograph the slides.

### 2.6. Statistical Analysis

The study replicated the work and reported results as mean ± SEM, with statistical analysis using ANOVA and Tukey's test. SPSS version 22 was used for the analysis, with significance at *P* < 0.001. The diagrams were created in Microsoft Excel 365. The study used histological analysis and evaluated mean rank with the Kruskal–Wallis test. Differences were analyzed with the Mann–Whitney *U* test with significance at *p* < 0.05.

## 3. Results

### 3.1. Measurement of IL-8 Cytokine Level

**Figure 1:**
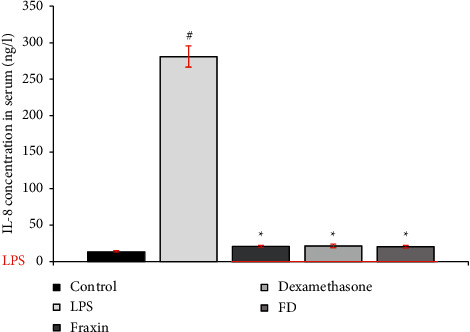
The level of IL-8 in LPS-induced mice serum after 24 hours. Treatment with fraxin, dexamethasone, and fraxin + dexamethasone (FD); data are presented as mean ± SEM; ^*∗*^highly significant *P* < 0.001 in comparison to LPS alone, and ^#^highly significant *P* < 0.001 in comparison to control, *n* = 10. The red line indicates groups injected with LPS (5 mg/kg), 1 hour after treatment.

Using a Shanghai YL Biont ELISA kit, the IL-8 level was quantified in the serum of treated mice. Fraxin and fraxin + dexamethasone (FD) activity was assessed in LPS-induced mice pretreated one hour before LPS induction.  Figure 1 shows that IL-8 levels were significantly reduced in treatment groups almost to the same degree (*p* < 0.001), and the percentage of inhibition of interleukin-8 for fraxin, dexamethasone, and FD was 93%, 92.4%, and 93%, respectively, compared to the LPS-only control.

### 3.2. Histopathological Analysis

**Figure 2:**
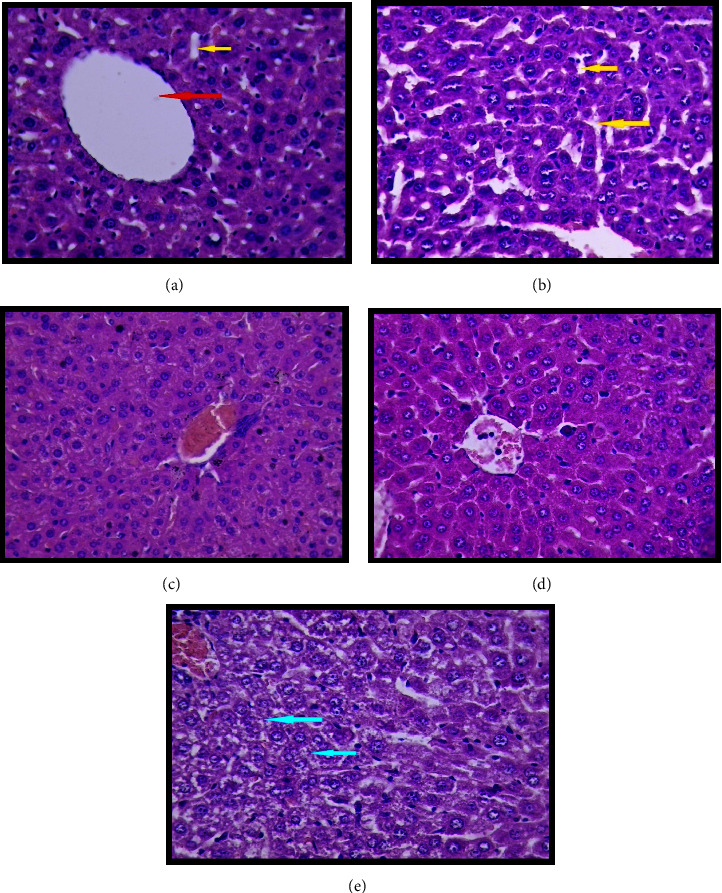
The histopathology of the liver (a). The control group liver section showing a normal structural appearance, central vein (red arrow), and sinusoids (yellow arrow), (b) lipopolysaccharide (LPS) only group liver section showing sinusoidal dilatation (yellow arrow), (c) (fraxin + LPS) near normal appearance of hepatocytes, (d) (DEX + LPS) liver section showing near-normal appearance, central vein, and threads of hepatocytes, and (e) (FD + LPS) liver section showing depletion of glycoprotein (blue arrow), hematoxylin and eosin, H&E stain, 40X.

**Figure 3:**
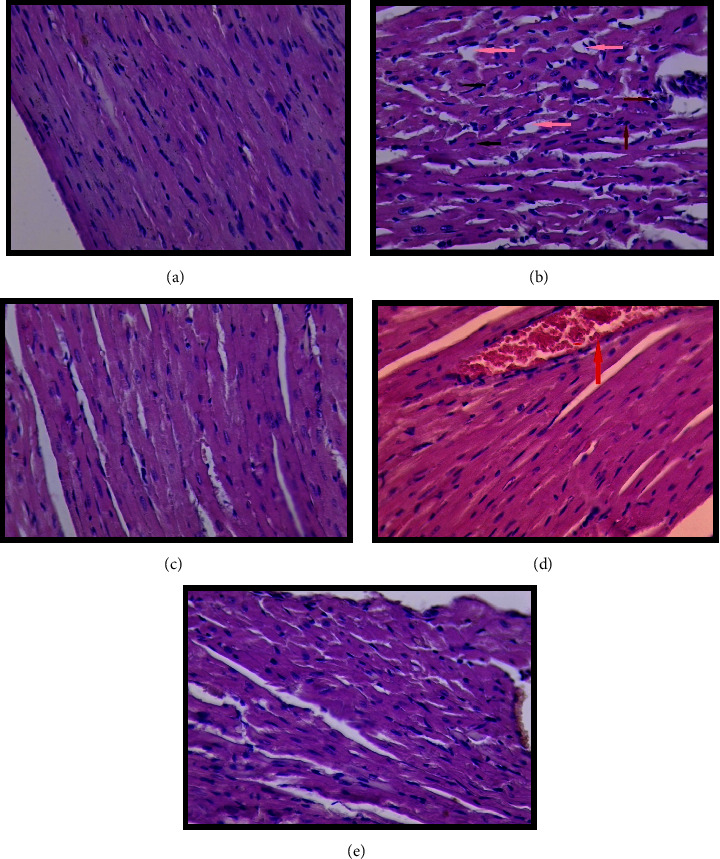
Histopathology of heart section. (a) Control group showing normal cardiac muscle fiber, central nucleus with presence of henle space, (b) lipopolysaccharide (LPS) only group showing dispersed necrosis of cardiac muscle fiber (black arrow) with mild inflammatory cell infiltration (red arrow), dilatation of Henle space (pink arrow), (c) (fraxin + LPS) showing near-normal appearance, (d) (DEX + LPS) heart section showing near normal appearance with mild congestion (red arrow), and (e) (FD + LPS) heart section showing near normal appearance. Hematoxylin and eosin, H&E stain, 40X.

For all treatment groups, histopathological findings for the liver are shown in Figure 2 and for heart in Figure 3, and the control group that received NS only is shown in Figure 2(a); the liver section shows a normal appearance of central vein and sinusoids.  Figure 3(a) shows the normal cardiac muscle fiber of the heart section with a central nucleus and presence of Henle space; histoscore was zero for all tissue sections, which indicates no damage at all in this group.

In contrast, the LPS-induced group in Figure 2(b), liver, Figure 3(b), heart, in Figure 2(b) showed sinusoidal dilatation in the liver section, while Figure 3(b) shows dispersed necrosis of the cardiac muscle fiber with atrophy of other cells, mild inflammatory cell infiltration, and dilatation of the Henle space.

The treatment groups significantly reduced tissue damage compared to the LPS-induced group (*p* < 0.05) in all tissue sections.

In liver sections, groups (Figures 2(c) and 2(d)) treated with (fraxin + LPS) and (DEX + LPS) showed a near-normal appearance of the hepatocytes, central veins, and sinusoids. In contrast, the group treated with (FD + LPS) (Figure 2(e)) showed glycoprotein depletion. In heart sections (Figure 3(c)), (fraxin + LPS) showed the most cardioprotective effect against the LPS-only group.

**Figure 4:**
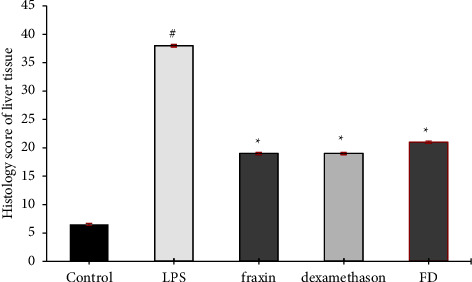
The histopathological scoring for all liver tissue treatment groups, with data presented as mean rank ± SEM. ^*∗*^Treatment groups demonstrated statistical significance at *p* < 0.05 compared to the lipopolysaccharide LPS group and ^#^significant at (*p* < 0.05) for LPS compared to the control, *n* = 5.

**Figure 5:**
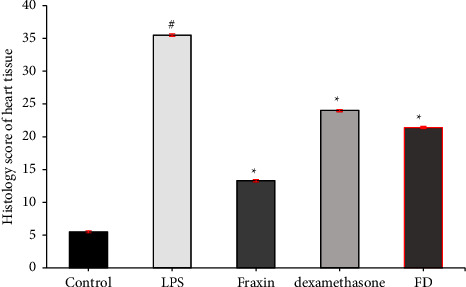
The histopathological scoring for all heart tissue treatment groups, with data presented as mean rank ± SEM. ^*∗*^Treatment groups demonstrated statistical significance at *p* < 0.05 compared to lipopolysaccharide, LPS group, and ^#^significant at (*p* < 0.05) for LPS compared to control, *n* = 5.

Figures 4 and 5Show the histopathological score represented as a mean rank±SEM for all treatment groups for liver tissue and heart tissue, respectively.

## 4. Discussion

In the current study, we tested the activity of fraxin, dexamethasone, and their fraxin + dexamethasone (FD) combination on a model of systemic inflammation developed by injecting LPS intraperitoneally (5 mg/kg) once. The observed inhibitory percentages for the interleukin-8 were 93% for fraxin, 92.4% for dexamethasone, and 93% for FD, compared to the control group that was exposed solely to LPS. All treatments dramatically reduced IL-8 levels in mouse serum; this effect could be attributed to the broad pharmacological action of fraxin, which included antiinflammatory, antioxidant, immune-modulatory, antiaging, anticancer, and antibacterial properties [12]. The present study builds on a previous research that demonstrated the ability of fraxin to act as an antagonist to LPS-triggered cytokine release. Li and his colleagues conducted two separate studies that established potential mechanisms through which fraxin may facilitate this biological response. Specifically, their findings suggested that fraxin may achieve its effects by manipulating signaling pathways associated with inflammatory cells, such as the NF-*κ*B pathway and NLRP3. Furthermore, it was found that the concentrations of tumor necrosis factor-*α* and interleukin-6 in serum and selected anatomical structures, specifically the lung and liver, were decreased [17].

In histopathological aspects, the results of this investigation indicate that the administration of fraxin, dexamethasone, and their combination successfully mitigated the deleterious effects of lipopolysaccharide (LPS) in various organs. From the previous literature, the administration of fraxin has been shown to mitigate histopathological damage to the liver resulting from cisplatin, evidenced by a reduction in necrosis of coagulation, hydropic degeneration, sinusoidal dilatation, and hyperemia. [18]. Also, fraxin exhibited a notable protective effect in countering liver damage induced by CCl_4_, resulting in a dose-dependent reduction in pathological scores [11].

The ethyl acetate fraction of *Fraxinus xanthoxyloides*, denoted as FXE, demonstrated a certain degree of cardioprotective effects at a low dose of 150 mg/kg compared to CCl_4_. On the contrary, the high dosage of FXE at 300 mg/kg and silymarin exhibited a protective efficacy comparable to that of the control after CCl_4_ administration. At both doses administered, FXE did not induce any histological alterations in cardiac tissues, indicating that it does not have a discernible impact on normal tissue morphology [19].

The administration of corticosteroids is associated with specific considerations, including decreased immune response and increased susceptibility to infections. In contrast, the use of corticosteroids for recovery from cytokine storms related to viral infection has shown promise, and it is important to consider that their immunosuppressive effects could potentially exacerbate viral load and increase susceptibility to secondary infections [20]. From the current study, we can recommend that fraxin alone and in combination with a low dose of dexamethasone may be a safer option since it will not affect the normal function of the immune system.

## 5. Conclusion and Recommendations

The findings of our investigation revealed that fraxin, dexamethasone, and the combination FD exhibit considerable efficacy in reducing levels of interleukin-8 (IL-8) in vivo in mice subjected to lipopolysaccharide (LPS) challenge through modulation of various signaling pathways. Significantly, from the previous literature, these compounds inhibit TLR-mediated NF-*κ*B signaling. Histopathological analysis showed that all interventions employed in this investigation reduced LPS-induced severe liver and cardiac damage. Histopathological scores exhibited a marked reduction compared to the group receiving only LPS treatment. Concurrent use of fraxin and dexamethasone in LPS-induced mice did not result in discernible superiority of either agent individually, as evidenced by equivalent quantification of IL-8 and findings of the comparable histopathological analysis in both the liver and the heart. Still, future studies could consider fraxin an anticytokine storm agent. Additional research on fraxin and its combinations with other phytochemicals is essential to evaluate the effect of these agents on antiinflammatory cytokines such as IL-10; different concentrations and different combinations can be considered in future work and research to find the precise way these drugs work and determine the potential applicability of these agents in the management of hyperinflammatory conditions, such as cytokine storm, in clinical practice.

The present study encountered certain limitations that must be acknowledged. Each treatment and the combination were evaluated using a single concentration. It should be noted that the limited sample size of animal groups and the specific location of blood withdrawal may significantly impact cytokine measurement levels. Additionally, the sampling timingcan increase the chance of measurement variability.

## Figures and Tables

**Table 1:** The chemicals and reagents used in this study.

Chemical/reagent	Provider
Fraxin purity (>98%)	Hangzhou Hyper Chemicals Limited, China
Dexamethasone purity (>98%)	Hangzhou Hyper Chemicals limited, China
LPS (*Escherichia coli*, 055: B5)	Hangzhou Hyper Chemicals limited, China
Dimethyl sulfoxide, DMSO	Thomas Baker, India
Mouse IL-8 ELISA kit	Shanghai YL Biont, China
10% formalin, formaldehyde	PanReac. AppliChem., Spain
Diethyl ether	Thomas Baker, India
N.S. 0.9% normal saline	PSI, Saudi Arabia

**Table 2:** An overview of the treatments employed, including details on dosage and route of administration.

Treatment group	Dosage	Route of administration	*n* = 10
LPS	5 mg/kg	Intraperitoneally, i.p.	10
Normal saline, N.S. (control)	1 ml/kg	Intraperitoneally, i.p.	10
Fraxin	120 mg/kg	Intraperitoneally, i.p.	10
Dexamethasone	5 mg/kg	Intraperitoneally, i.p.	10
Fraxin + dexamethasone (FD)	60 mg/kg + 2.5 mg/kg	Intraperitoneally, i.p.	10

## Data Availability

The experimental data used to support the findings of this study may be released upon application to the corresponding author, nadasshaker@gmail.com.

## Abbreviations

ANOVA: Analysis of variance
ARDS: Acute respiratory distress syndrome
CCl_4_: Carbon tetrachloride
COVID: Coronavirus disease of 2019
CXC: Chemokine group
Dexa: Dexamethasone
DMSO: Dimethyl sulfoxide
ELISA: Enzyme-linked immunosorbent assay
FD: Fraxin + dexamethasone treatment group
FXE: *Fraxinus xanthoxyloides* ethyl acetate fraction
H&E: Hematoxylin and eosin
HRP: Horse radish peroxidase
IL-1*β*: Interleukin-1 beta
IL-6: Interleukin-6
IL-8: Interleukin-8
i.p: Intraperitoneal
LPS: Lipopolysaccharide
mAb: Monoclonal antibody
MAPKs: Mitogen-activated protein kinase
NF-*κ*B: Nuclear factor kappa B
ng/L: Nanogram per liter
NLRP3: Nucleotide-binding domain, leucine-rich–containing family, pyrin domain–containing-3
nm: Nanometer
NS: Normal saline
OD: Optical density
PPAR*γ*: Peroxisome proliferator-activated receptor *γ*
SARS-CoV-2: Severe acute respiratory syndrome coronavirus 2
SEM: Standard error of the mean
SPSS: Statistical Package for the Social Sciences
TLR4: Toll-like receptor 4
TNF*α*: Tumor necrosis factor *α*.
